# Endospore appendages enhance adhesion of *Bacillus cereus sensu lato* spores to industrial surfaces, modulated by physicochemical factors

**DOI:** 10.1128/aem.00944-25

**Published:** 2025-10-07

**Authors:** Unni Lise Albertsdottir Jonsmoen, Jennie Ann Allred, Dmitry Malyshev, Jonas Segervald, Magnus Andersson, Marina Elisabeth Aspholm

**Affiliations:** 1 Department of Paraclinical Sciences, Faculty of Veterinary Medicine, Norwegian University of Life Sciences (NMBU) 56625, Ås, Norway; 2 Department of Physics, Umeå University 8075, Umeå, Sweden; 3 Umeå Centre for Microbial Research 504517 , Umeå, Sweden; Anses, Maisons-Alfort Laboratory for Food Safety, Maisons-Alfort, France

**Keywords:** colonization, ENA, adhesion, endospore, *Bacillus cereus*

## Abstract

Spores of species belonging to the *Bacillus cereus sensu lato* (s.l.) group are common contaminants in food processing environments due to their ability to adhere to surfaces and resist cleaning procedures. These spores are equipped with pilus-like endospore appendages (ENAs), which are believed to promote surface adhesion. We investigated the role of ENAs in spore adhesion to abiotic surfaces using a wild-type (WT) *Bacillus paranthracis* strain and isogenic mutants lacking ENAs or an intact exosporium. WT spores expressing both short and long ENAs (S+L+) adhered significantly more to stainless steel (SS) and polypropylene (PP) compared to bald spores (S–L–) and spores of an exosporium-deficient mutant (Δ*exsY*), whereas adhesion to polystyrene (PS) and glass was not significantly affected by the presence of ENAs. The Δ*exsY* mutant also showed the lowest adhesion across all tested surfaces, a pattern similarly observed for vegetative cells. The strongest adhesion to PP was observed when both fiber types were present. A clear trend also emerged: on PP, WT remained adhered for at least an hour, while bald spores tended to detach within that time. Under saline conditions and at different pH levels, bald spores adhered strongly to SS. However, in the presence of a non-ionic surfactant or a concentrated protein solution, WT spores adhered more. Our results highlight the crucial role of ENAs in *B. cereus* spp. spore adhesion to industrially relevant surfaces, providing mechanistic insight into spore persistence. These insights support the design of surface treatments to prevent contamination, spoilage, and foodborne illnesses.

**IMPORTANCE:**

Bacteria belonging to the *Bacillus cereus sensu lato* group represent a persistent challenge in food production due to their highly resilient endospores (spores), which withstand cleaning, disinfection, and food processing. Understanding spore adhesion is essential for designing effective surface treatments that reduce chemical use, enhance food safety and quality, and minimize environmental impact. This study underscores the important role of endospore appendages (ENAs) in spore adhesion to common materials in food processing and laboratory environments. Wild-type spores expressing both S-ENA and L-ENA adhered significantly more than mutants lacking ENAs or the exosporium, highlighting ENAs as potential targets for disrupting spore adhesion. Time-dependent adhesion assays on polypropylene revealed strong, sustained attachment by wild-type spores, contrasting with weaker, transient adhesion by ENA-depleted mutants. These findings offer valuable insights into *B. paranthracis* spore adhesion dynamics, guiding the development of tailored cleaning protocols to improve contamination control and sustainability.

## INTRODUCTION

Spore-forming bacteria belonging to the *B. cereus sensu lato* (s.l.) group pose significant challenges to the food industry, frequently contaminating dairy products and other food items such as rice, pasta, spices, and vegetables (1). This group of bacteria produces a range of different degradative enzymes during growth in food, with many strains also producing toxins associated with foodborne illnesses (2, 3). The hygienic risk associated with these strains is amplified by their ability to produce highly resilient endospores, commonly referred to as “spores.” Spores can survive wet heat, including pasteurization, high pressure, desiccation, and other food processing steps that are intended to eliminate contaminating microorganisms (4). Moreover, they can form biofilms that are particularly challenging to eradicate once established. The protective extracellular matrix of the biofilm shields embedded cells and spores from cleaning agents and environmental stresses, while also periodically releasing them into the surroundings. This results in persistent contamination in food production environments, causing an ongoing threat to both product safety and quality (5). The presence of biofilm not only increases the risk of foodborne illnesses but also complicates cleaning and sanitization processes, leading to higher operational costs and waste of food due to suboptimal quality. In the food industry, food contact surfaces are often composed of stainless steel and plastic. Additionally, typical food and drink containers, as well as sample vials and disposable laboratory equipment used for contamination assessments, are often made of plastic and glass. Several studies have shown that *B. cereus* spp. spores adhere effectively to these materials, with some attributing their strong adhesion to the hydrophobic nature of the exosporium (6, 7). Moreover, surface appendages and low zeta potential are believed to significantly enhance the spores’ adhesiveness (8). However, there are conflicting findings in the literature. Other studies have not established the same link to spore surface hydrophobicity, instead attributing the strong adhesiveness of *B. cereus* s.l. spores to their surface appendages (9, 10). Earlier functional studies on *B. cereus* s.l. spore appendages and their role in adhesion to abiotic surfaces were primarily done by comparing spores with intact appendages to those from which these fibers had been mechanically removed (8, 11, 12), but these studies produced variable results: some reported enhanced adhesion associated with the appendages, while others found no significant correlation, or even suggested that the appendages might reduce adhesion under certain conditions (8–11, 13). These inconsistencies highlight a knowledge gap in our understanding of the mechanisms underlying spore adhesion and the functional role of their appendages.

**Fig 1:**
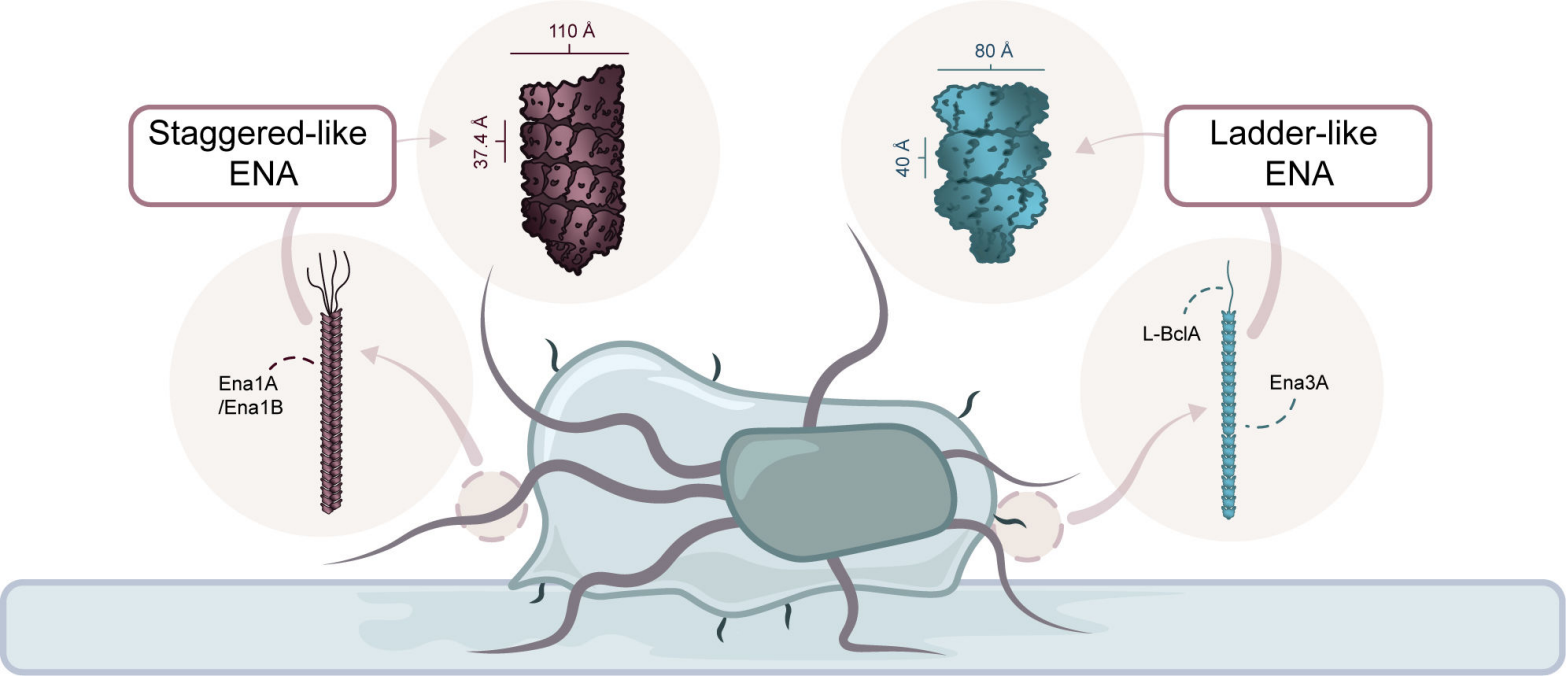
Schematic illustration of an endospore adhered to a surface, with both S-ENAs (red) and L-ENAs (blue) highlighted.

The structure and composition of endospore appendages (ENAs) remained unresolved for a long time, but technological advancements have enabled further research to unravel their characteristics and functions. Recent studies on the foodborne outbreak strain *Bacillus paranthracis* NVH 0075/95 (classified as *B. cereus sensu stricto* until 2022) have identified two distinct types of ENAs: the staggered S-ENA and ladder-like L-ENA (14). The S-ENA protrudes from the endospore coat and is 8–12 nm thick, 0.4–1.2 µm long and displays four to five filamentous fibrillae at its distal end (14, 15). This fiber is built of two major protein subunits, Ena1A and Ena1B, which self-assemble into a helical structure, as illustrated in Fig. 1. The L-ENA, which is thinner and shorter than the S-ENA, is attached to the exosporium layer, measures 2.5–3.5 nm in thickness, 0.2–1.6 µm in length, and is built of the major subunit Ena3A (15, 16). The L-ENA fiber also carries a single tip fibrillum at its distal end, which is composed of the collagen-like L-BclA protein, a member of the *Bacillus* collagen-like protein family (16). Recent findings by Jonsmoen et al. (17) suggest that L-BclA is also expressed at the end of the S-ENA fiber, forming one or more of its terminal fibrillae (17).

The recent discoveries about the structure and genetic origins of ENAs have facilitated the development of mutants lacking S- and/or L-ENAs, allowing for more detailed studies of their function(s) (17, 18). For example, both S- and L-ENAs have been shown to play key roles in spore-to-spore contact, leading to spore self-aggregation (17). Additionally, appendages, particularly L-ENAs, were found to enhance adhesion to glass surfaces (18). However, the role of ENAs in binding to common materials used in food production and laboratory settings, as well as the influence of chemical conditions on their adhesive properties, remains poorly understood.

In this study, we hypothesize that S- and L-type ENAs play a role in enhancing spore adhesion to abiotic surfaces commonly encountered in food production and laboratory environments. Using *B. paranthracis* NVH 0075/95 and isogenic mutants with distinct ENA phenotypes, we evaluated spore adhesion to stainless steel (SS), commonly used in food processing equipment, polystyrene (PS) and polypropylene (PP), prevalent in packaging and sampling materials (19), and glass, a material of central importance in laboratory environments. Additionally, we examined how the presence of salt (buffer solution), pH, and extended contact time influenced spore adhesion.

## RESULTS

### ENAs influence spore surface adhesion to stainless steel and polypropylene

The role of ENAs in adhesion to abiotic surfaces, SS, PP, PS, and glass, was assessed by comparing wild-type (WT) *B. paranthracis* NVH 0075/95 spores (S+L+) with bald spores lacking both short and long ENAs (S–L–). We also included spores lacking a complete exosporium due to deletion of the major exosporium protein gene, *exsY*, although some residual exosporium fragments remain. The Δ*exsY* mutant still produces S-ENAs as it is attached to the coat, while L-ENAs are absent as their anchoring is dependent on the exosporium surface (17, 18). To provide a broader comparison, adhesion of vegetative *B. paranthracis* cells was also measured. The vegetative cells carry flagella-like extensions (17) but do not express ENAs (14, 16). The morphology and genotypes of all strains used in this study are summarized in Table 1.

**TABLE 1:** Genotype and morphology of the strains used in this work

Name	Genotype	Spore surface-related phenotype^*a*^			Reference
		S-ENA	L-ENA	Exosporium	
S+L+ (WT)	WT	+	+	+	(20)
S+L–	Δ*ena3*	+	−	+	(16, 18)
S–L+	Δ*ena1ABC*	−	+	+	(14)
S−L− (bald)	Δ*ena1ABC* Δ*ena3*	−	−	+	(16, 18)
Δ*exsY*	Δ*exsY*	+	−	−	(18)

^*a*^ − denotes lack of the indicated ENA fiber, while + denotes its presence.

**Fig 2:**
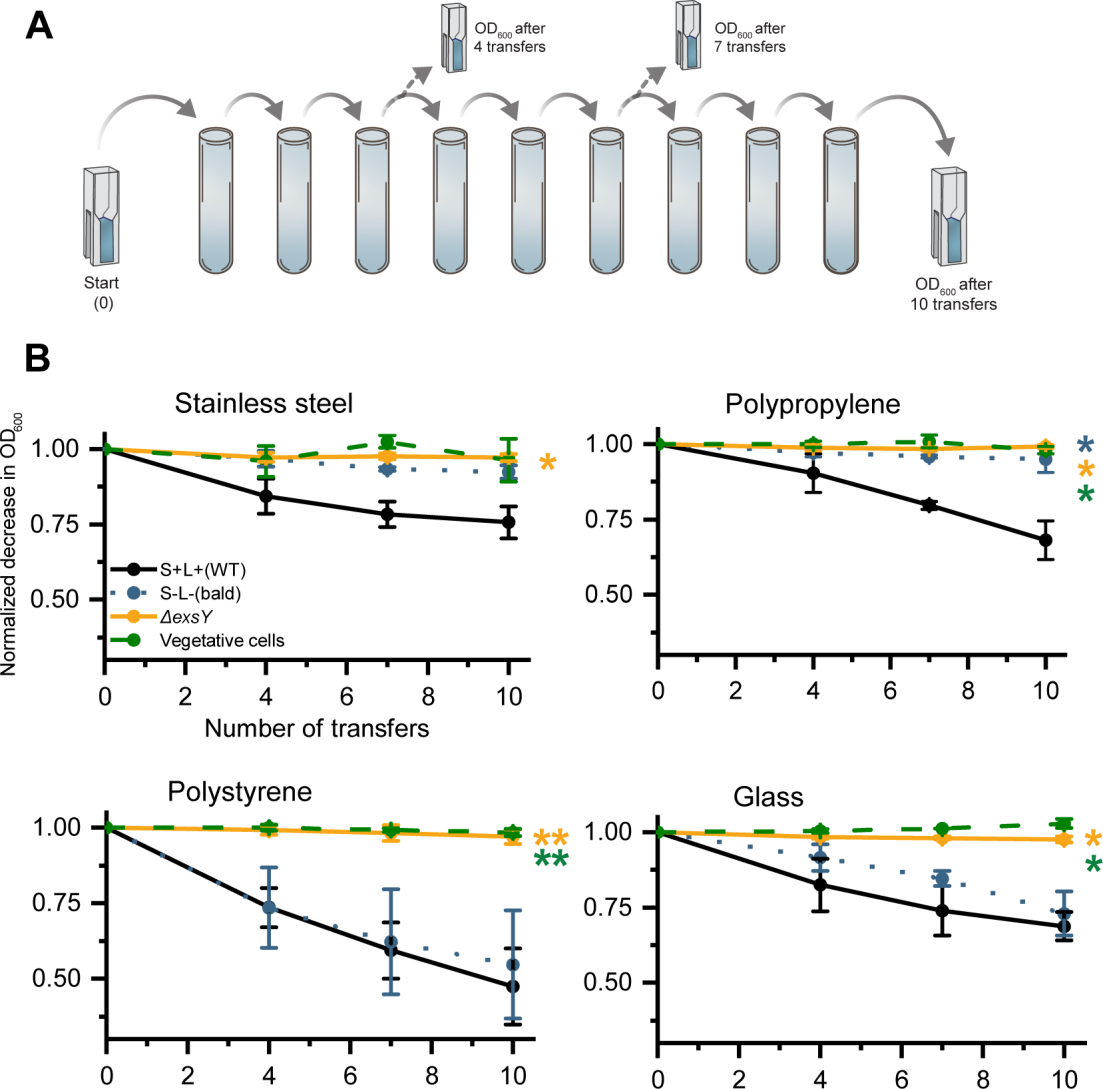
(**A**) Schematic illustration of the methodology for measuring spore and cell adhesion to stainless steel, polypropylene, polystyrene, and glass by loss of OD_600_ after 4 transfers, 7 transfers, and 10 transfers. (**B**) The measured adhesion of WT, bald, Δ*exsY* spores, and vegetative cells, on SS, PP, PS, and glass, revealed varied impact of ENAs across the different tested materials. The largest impact of ENAs was attributed to SS and PP. Exosporium-deficient spores and vegetative cells had the least adhesion efficiency for all materials tested. One asterisk (*P* ≤ 0.05) indicates significant difference, while two (*P* ≤ 0.01) and three (*P* ≤ 0.001) asterisks denote increasingly lower *P*-values.

The role of ENAs in spore adhesion to SS, PP, PS, and glass was evaluated by sequentially transferring spores and vegetative cells between vials or tubes and then measuring the reduction in OD_600_, which served as an indicator of spore loss due to adherence to the materials tested (Fig. 2A). The results demonstrated that ENA-mediated spore adhesion was surface-dependent (Fig. 2B). On SS, wild-type spores exhibited the highest adhesion efficiency, while both bald and Δ*exsY* spores showed significantly reduced adhesion compared to those of the WT strain (*P* = 0.0014 and *P* = 0.0003, respectively, after 10 transfers). A similar pattern was observed on PP, where WT spores adhered significantly more than both bald spores (*P* = 0.0004) and exosporium-deficient spores (*P* = 0.0002). In contrast, adhesion to PS and glass was equally efficient for WT and bald spores, suggesting that ENAs play a less prominent role in spore attachment to these surfaces. Nonetheless, Δ*exsY* spores again displayed the lowest adhesion on both glass (*P* = 0.0005) and PS (*P* = 0.0002) after 10 transfers, suggesting that an intact exosporium may contribute to adhesion independently of ENAs on certain surfaces.

Adhesion of vegetative cells to the same materials was also assessed. These cells showed minimal adhesion across all tested surfaces, with levels even lower than those of the Δ*exsY* spores (Fig. 2B). This underscores the limited adhesive capacity of vegetative cells and highlights the distinct contribution of the exosporium and ENAs to spore adhesion in *B. paranthracis*.

Overall, a clear trend emerged: WT spores adhered more efficiently than bald spores, Δ*exsY* spores, and vegetative cells on SS and PP. On PS and glass, however, WT spores only outperformed Δ*exsY* spores and vegetative cells, with no significant difference compared to bald spores. These findings underscore the complex, surface-specific role of ENAs in spore adhesion, suggesting that their contribution is strongly influenced by the physicochemical properties of the substrate.

### Limited role of surface and spore hydrophobicity in spore adhesion

**Fig 3:**
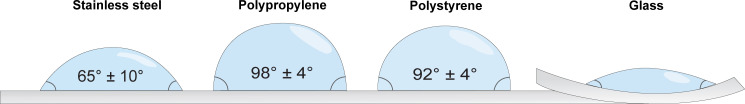
Contact angles were determined by measuring the angle formed between a water droplet and the different surface materials (SS, PP, PS, and glass). The resulting droplet profiles are presented as an outline for all materials. The contact angle for glass could not be determined due to the highly curved surface, which resulted in unreliable measurements. SS exhibited hydrophilic properties, while PP and PS were classified as hydrophobic.

Since ENAs significantly influenced spore adhesion to both SS and PP, as demonstrated by comparisons between wild-type spores and those lacking both S- and L-ENAs, we next investigated whether hydrophobicity could help explain these patterns. To assess potential differences in hydrophobicity between the materials, we performed sessile drop contact angle measurements using water as the test liquid. By measuring the angle formed between the liquid droplet and the surface material, we could determine if the surfaces were hydrophobic or hydrophilic. As seen in Fig. 3, both PP and PS exhibited contact angles greater than 90°, classifying these as hydrophobic. In contrast, SS showed a contact angle below 90°, indicating a hydrophilic surface. Accurate measurement of wettability on glass was not possible due to the curvature of the substrate; therefore, only its outline is included in the figure. Raw data are available in the supplemental material (Data for contact angle and surface roughness measurements, Fig. S1 and Table S1).

Because of the varying wettability of the materials, isolating the specific effect of hydrophobicity on spore surface adhesion was not possible. Therefore, to quantify adhesion under controlled conditions, we adapted the spore transfer setup to 96-well microtiter plates with uniform well dimensions and measured absorbance at 600 nm. This setup allowed us to compare spore adhesion on hydrophilic surfaces (PS-treated) with a hydrophobic surface (PS-untreated) (21, 22). The two types of PS materials were selected to provide a controlled baseline for surface hydrophobicity, while minimizing the potential influence of other factors, e.g., surface roughness and hardness.

**Fig 4:**
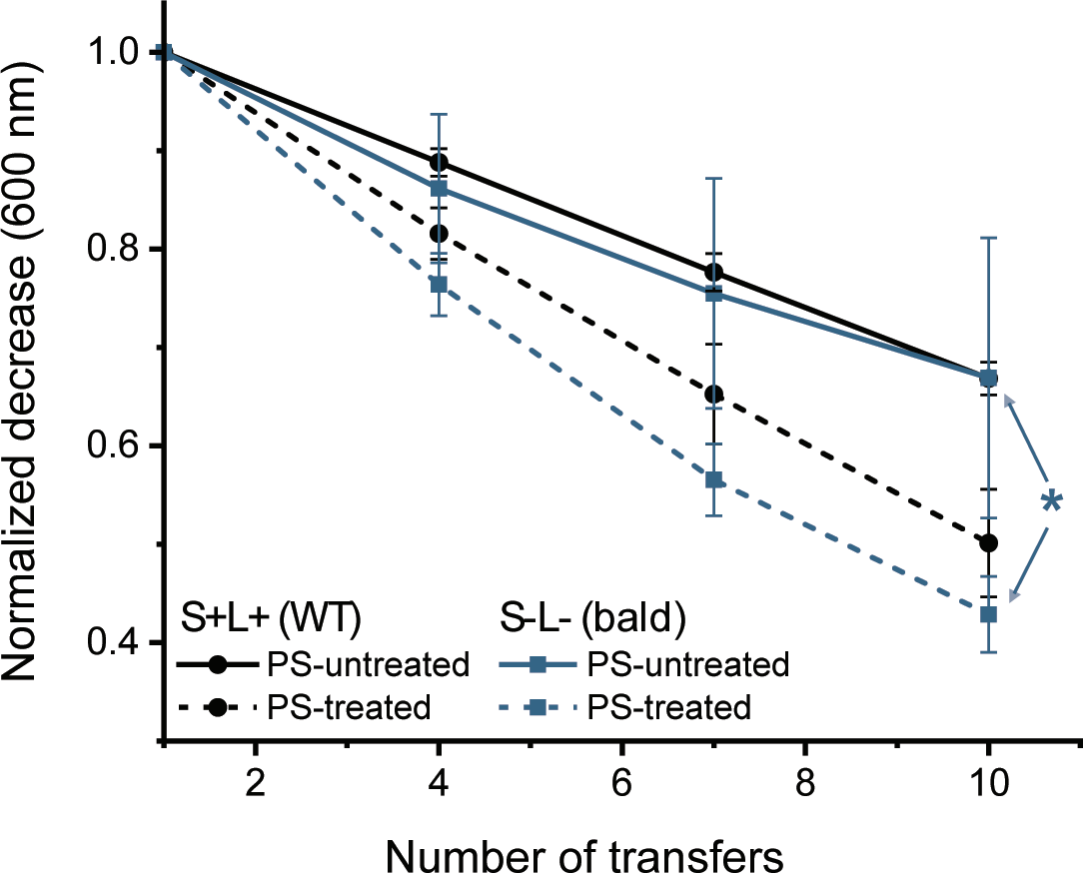
Adhesion efficiency of WT and bald spores after 10 transfers was assessed on polystyrene (PS) surfaces with different treatments: untreated (hydrophobic) and treated (hydrophilic). The results showed that WT (S+L+) spores exhibited no significant preference between the two surface types based on the average adhesion from three independent batches. In contrast, bald (S–L–) spores adhered significantly more to the hydrophilic surface, as indicated by a single asterisk denoting statistical significance (*P* ≤ 0.05). A significant difference was also observed between WT spores on the treated surface and bald spores on the untreated surface; however, this comparison is not shown in the figure, as it falls outside the primary scope of this study.

The results presented in Fig. 4 show that WT spores do not preferentially adhere to either the hydrophobic or hydrophilic surface, as no statistically significant difference was observed after the 10th transfer (*P* > 0.1). Furthermore, the presence of ENAs did not appear to influence adhesion, as both WT and bald spores adhered with similar efficiency to both surfaces. While showing a similar trend to the WT spores, the bald spores displayed significantly higher adhesion to the hydrophilic surface compared to the hydrophobic substrate after 10 transfers. However, the *P*-value is close to the significance threshold of 0.05 (*P* = 0.0470) and should be interpreted with caution. Consequently, the inherent wettability of the substrate does not appear to be the primary factor influencing ENA-mediated spore adhesion. Instead, the spores’ intrinsic properties, potentially mediated by ENAs, seem to play a more decisive role.

**Fig 5:**
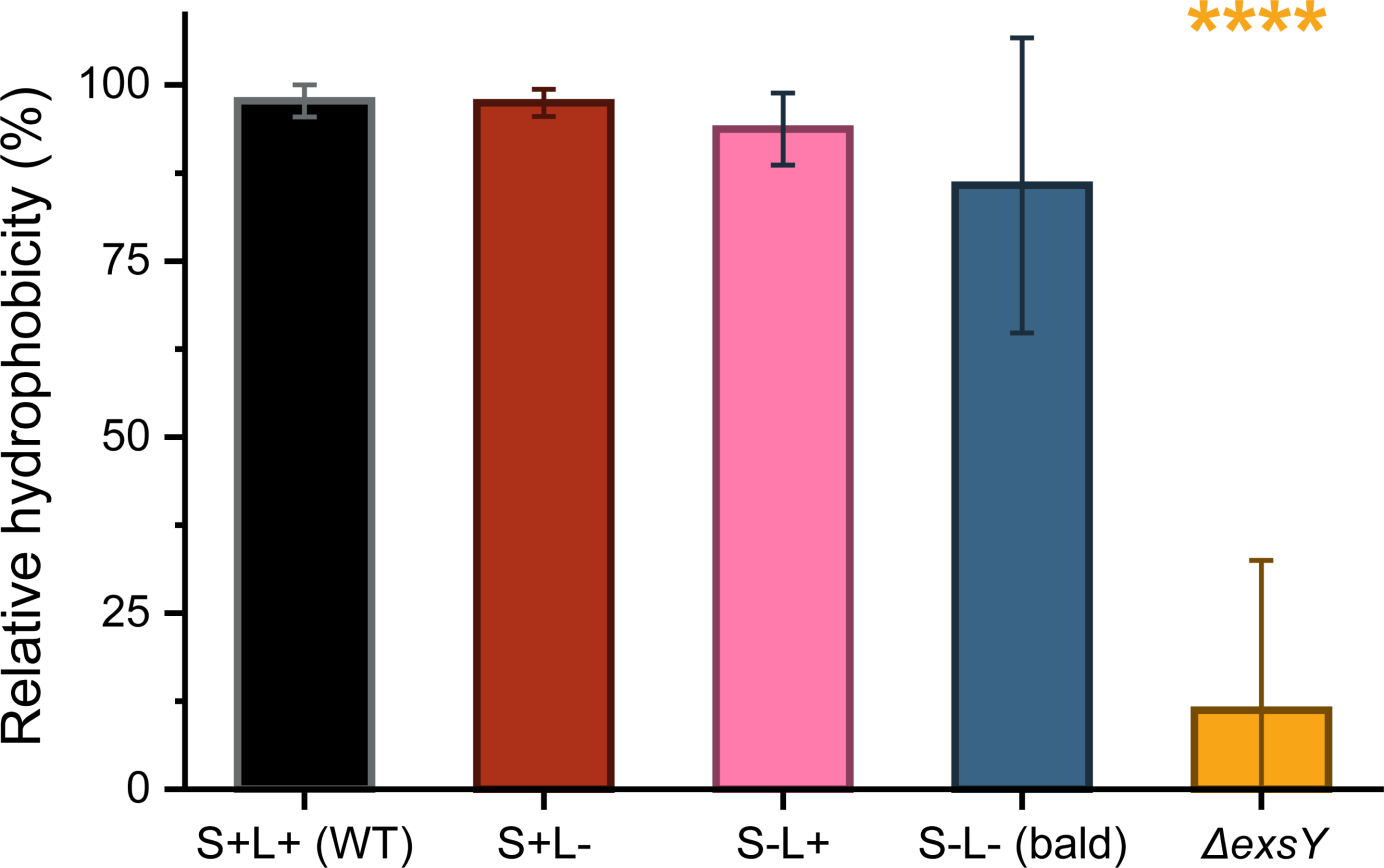
Relative hydrophobicity of WT spores was compared to various isogenic mutant spores, using the MATH test. The results show that removal of the ENAs does not significantly affect the hydrophobicity of the spores. However, the exosporium-deficient spores exhibited the lowest relative hydrophobicity compared to WT spores, with a statistical significance *P* < 0.0001 as denoted by the four asterisks in the figure. The results are based on three biological replicates.

We also examined whether ENAs affect spore surface hydrophobicity by performing a microbial adhesion to hydrocarbon (MATH) test. To assess the individual contributions of S-ENAs and L-ENAs to hydrophobicity, two additional isogenic mutants were also included in the experiment: one expressing only S-ENAs (S+L−) and the other only L-ENAs (S−L+). The results showed no significant differences in hydrophobicity between ENA-depleted spores and WT spores (Fig. 5) although S−L− spores displayed a high standard deviation (16%). In contrast, the exosporium-deficient mutant exhibited significantly lower hydrophobicity compared to both WT and ENA-depleted spores (*P* < 0.0001), indicating that the exosporium, rather than ENAs, plays a key role in modulating spore surface hydrophobicity.

In conclusion, neither surface nor spore hydrophobicity appear to influence ENA-mediated adhesion to the tested substrates. To better understand why ENAs promote strong adhesion to certain materials but have a weaker effect on others, we further investigated the intrinsic properties of the materials themselves.

### Stainless steel features large surface structures, while glass exhibited overall lowest roughness

To better understand how the inherent properties of the tested substrate influence spore adhesion, a detailed material characterization was performed using scanning electron microscopy (SEM), tensiometer, profilometer, and atomic force microscopy (AFM). This in-depth analysis provides insights into the factors driving preferential spore adhesion to specific substrates.

**Fig 6:**
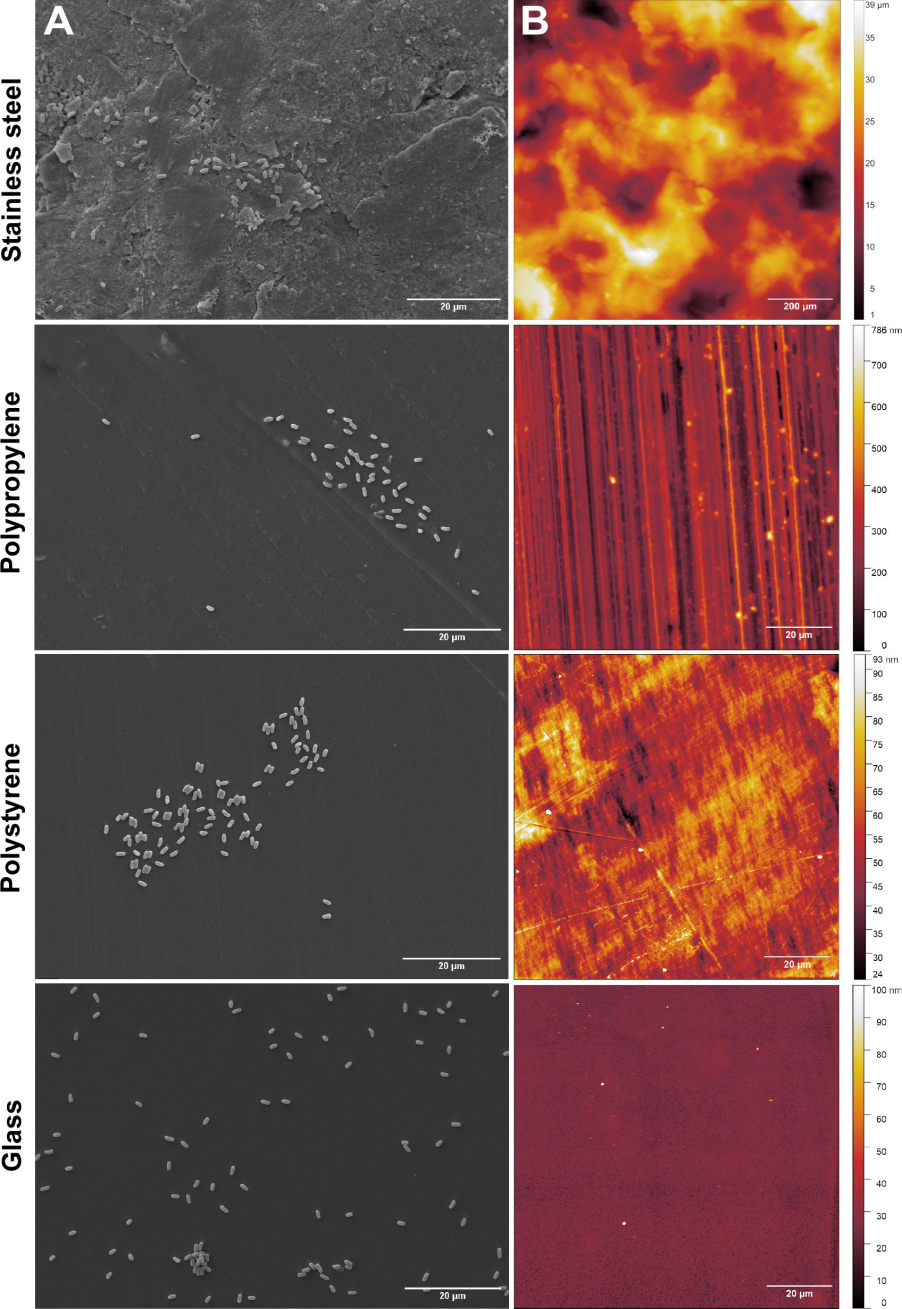
(**A**) Scanning electron micrographs showing spores adhered to stainless steel, polypropylene, polystyrene, and glass surfaces. The SS surface showed pronounced irregularities, in contrast to the glass surface, which appeared smoothest overall. A scale bar of 20 µm is shown for all materials. (**B**) Height maps were generated using a profilometer for stainless steel and AFM for PP, PS, and glass. The range of surface heights is displayed on the right side of each map, presented in µm for SS and in nm for PP, PS, and glass.

As shown in Fig. 6A, the SEM micrographs revealed that the PP, PS, and glass surfaces were relatively smooth and uniform, with minimal irregularities. In contrast, the SS surface appeared highly textured, characterized by prominent grooves and ridges. To quantify these differences in surface topography, AFM and profilometry were performed. Surface scanning with a sharp probe revealed that glass had the lowest roughness (5 nm ± 2 nm), followed by PS (29 nm ± 16 nm), PP (92 nm ± 20 nm), and SS (7 µm ± 0.4 µm), which displayed the highest roughness as determined by root mean square (RMS) roughness measurements. Surface topography was also visualized as height maps in Fig. 6B, where darker regions represent lower areas and brighter regions indicate elevated areas. These visualizations further supported the calculated RMS roughness, confirming that SS had the roughest surface while glass had the smoothest. Additionally, the surface ratio (SR) was calculated to assess how surface texture contributes to an increase in actual surface area relative to the scanned area. Both glass and PS exhibited surface ratio values close to 1, indicating minimal surface expansion due to texture. In contrast, SS and PP showed slightly elevated surface ratio values (>1), suggesting a larger increase in actual surface area because of surface roughness. Lastly, autocorrelation analysis was performed to further understand the repetitive distribution of the grooves and ridges on each material surface. A Fourier transform was used to identify dominant periodic features independent of direction, while both vertical and horizontal autocorrelations provided insight into overall surface patterns (details are provided in Tables S2 to S5). Overall, SS exhibited larger-scale periodic features, as indicated by both Fourier and spatial autocorrelations analyses, whereas PP, PS, and glass displayed finer more closely spaced surface patterns. Interestingly, as seen on the height map, PP also exhibited a pronounced directional roughness.

### Synergistic effect of S-ENAs and L-ENAs essential for spore adhesion to polypropylene

**Fig 7:**
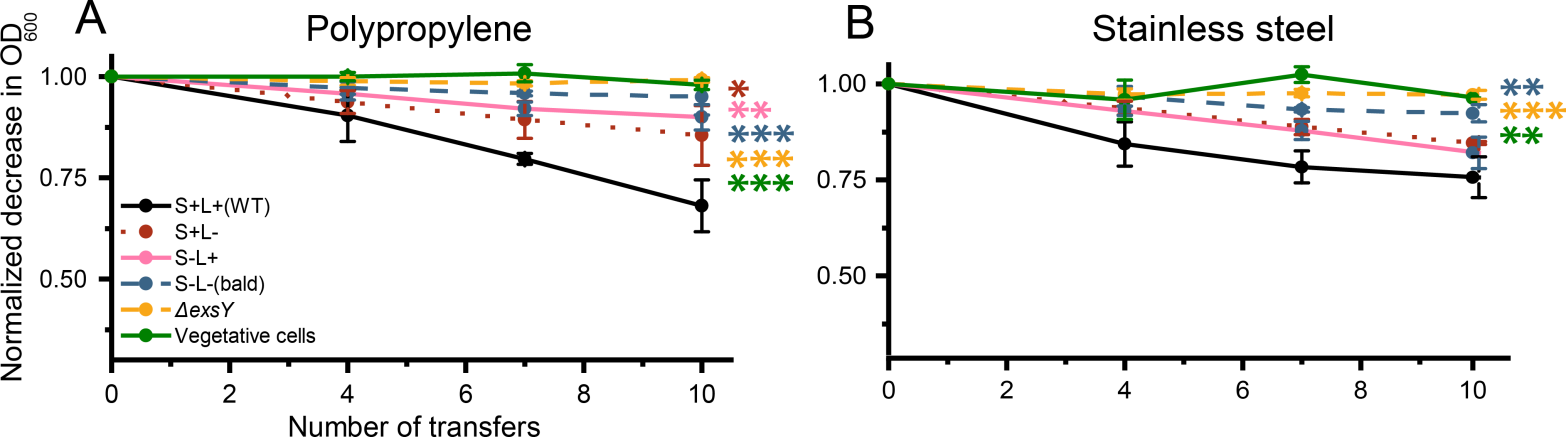
Loss of OD_600_ during sequential transfer of spores, with and without appendages, as well as isogenic mutant strains expressing only S-ENA (S+L−) or only L-ENA (S−L+), measured between PP tubes (**A**) and SS coupons (**B**). (**A**) After 10 transfers on PP, spores lacking either S-ENAs or L-ENAs showed significant reduction in adhesion compared to WT spores, indicating a collaborative role of both appendage types in achieving optimal adhesion. (**B**) In contrast, no significant differences were observed between WT spores and spores of either mutant strain on SS, suggesting that the presence of either S-ENA or L-ENA alone is sufficient to mediate effective adhesion to this surface. One asterisk (*P* ≤ 0.05) indicates a significant difference, while two (*P* ≤ 0.01) and three (*P* ≤ 0.001) asterisks represent increasingly stronger levels of statistical significance.

Recognizing the importance of ENAs in spore adhesion, we further investigated the specific contributions of each appendage type using the two isogenic mutant strains S−L+ and S+L−. For comparison, WT spores, bald spores, exosporium-deficient spores, and vegetative cells were also included in the analysis. PP and SS were selected as substrates, as the previous experiment (Fig. 2B) showed a significant difference in adhesiveness between WT and bald spores on these materials. Consistent with earlier results, a significant difference in adhesion to PP was observed between WT spores, bald spores, exosporium-deficient spores, and vegetative cells, with WT spores showing the highest adhesion after 10 transfers (Fig. 7). Additionally, both S+L− and S−L+ spores showed significantly reduced adhesion compared to WT spores (*P* = 0.0114 and *P* = 0.0031, respectively). These findings indicate that both S-ENAs and L-ENAs are necessary for spores to adhere effectively to polypropylene surfaces. Similarly, on SS, WT spores adhered significantly more than bald spores, exosporium-deficient spores and vegetative cells after 10 transfers. However, in contrast to the results on PP, no significant difference was observed between WT, S+L− and S−L+ spores on SS. This suggests that, on SS, the presence of either S-ENA or L-ENA alone is sufficient to achieve an adhesion efficiency comparable to WT spores.

### ENAs may be crucial for maintaining adhesion to polypropylene over time

**Fig 8:**
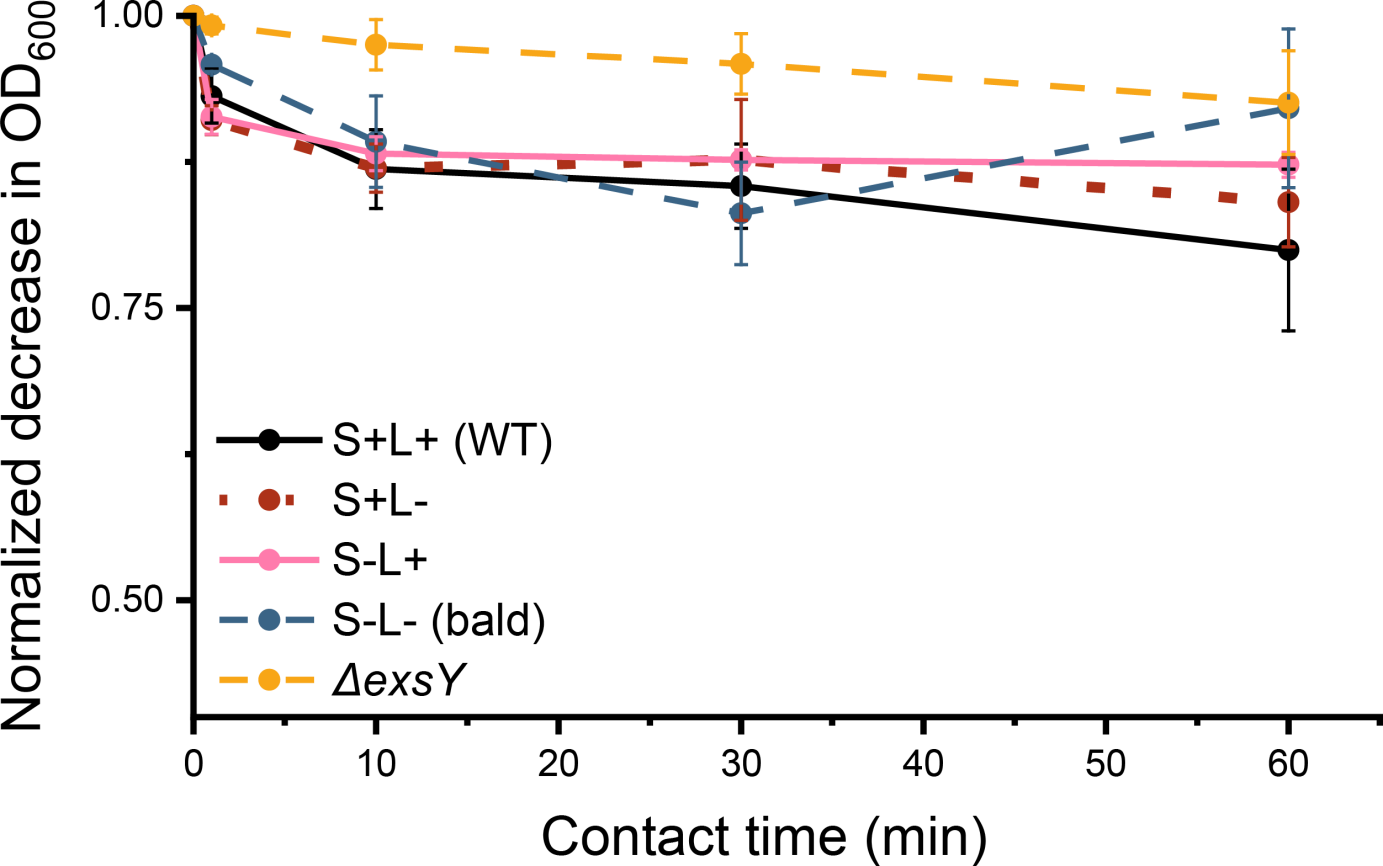
Adhesion of WT and isogenic mutant spores to PP was assessed at various incubation time points (1 min, 10 min, 30 min, and 60 min). The results showed that bald spores exhibited transient adhesion efficiency, with a peak at 30 min followed by a decline after 60 min. In contrast, WT spores maintained stable adhesion over time. Data represent three biological replicates. No statistically significant differences were detected by one-way ANOVA performed at 60 min with WT as control.

Previous experiments demonstrated substrate-dependent variations in spore adhesion, with ENAs playing a critical role, particularly on PP and SS. These measurements were taken over relatively short time periods, typically only a few seconds per transfer step. In industrial environments, spores are exposed to both continuous and intermittent flows (stop and go), resulting in varying contact times on the abiotic surfaces. To understand the effect exposure time on spore adhesion, we conducted an extended incubation experiment using PP tubes, which also allowed us to also implement rotation to emulate fluid conditions as observed in the food industry. WT and mutant spores were incubated and continuously rotated for 1, 10, 30, and 60 min. Adhesion was quantified by measuring the reduction in OD_600_ of the spore suspension at each time point (Fig. 8).

The results revealed a clear pattern of adhesion dynamics over time. WT spores, bald spores, and those expressing either S-ENA or L-ENAs maintained high levels of adhesion to the polypropylene surface after 1, 10, and 30 min of contact. In contrast, the exosporium-deficient mutant exhibited consistently weak adhesion at all points, underscoring the importance of the exosporium and its associated appendages in mediating stable surface interactions. Bald spores also displayed a more transient adhesion profile. Their adhesion peaked at 30 min, suggesting an initial ability to bind effectively to the PP surface. However, by the 60-min mark, their adhesion level decreased sharply, aligning with the level observed for the exosporium-deficient mutant. This significant drop in adhesion indicates that bald spores rely on transient binding mechanisms, which are insufficient for maintaining long-term attachment to PP surfaces. Although statistical significance could not be confirmed at the 30- or 60-min time points due to large standard errors, the observed trend supports the conclusion that ENAs play a critical role in maintaining spore adhesion over time, particularly on substrates such as PP.

### Ionic concentration impacts spore adhesion to stainless steel

Previous work demonstrated that the presence of salt in a spore suspension inhibits appendage-mediated spore-spore interactions (17). To investigate if similar effects occur in spore adhesion to abiotic surfaces, we examined the influence of salt and other relevant factors in the liquid environment. Spores were suspended in phosphate-buffered saline (PBS) to simulate elevated ionic strength, in NaOH and HNO_3_ to model chemical conditions during disinfection and cleaning-in-place procedures, in Tween-20 to represent high concentrations of non-ionic surfactants, and in bovine serum albumin (BSA) to mimic environments with increased protein content. SS was used as the sole surface due to its relevance in industrial settings.

**Fig 9:**
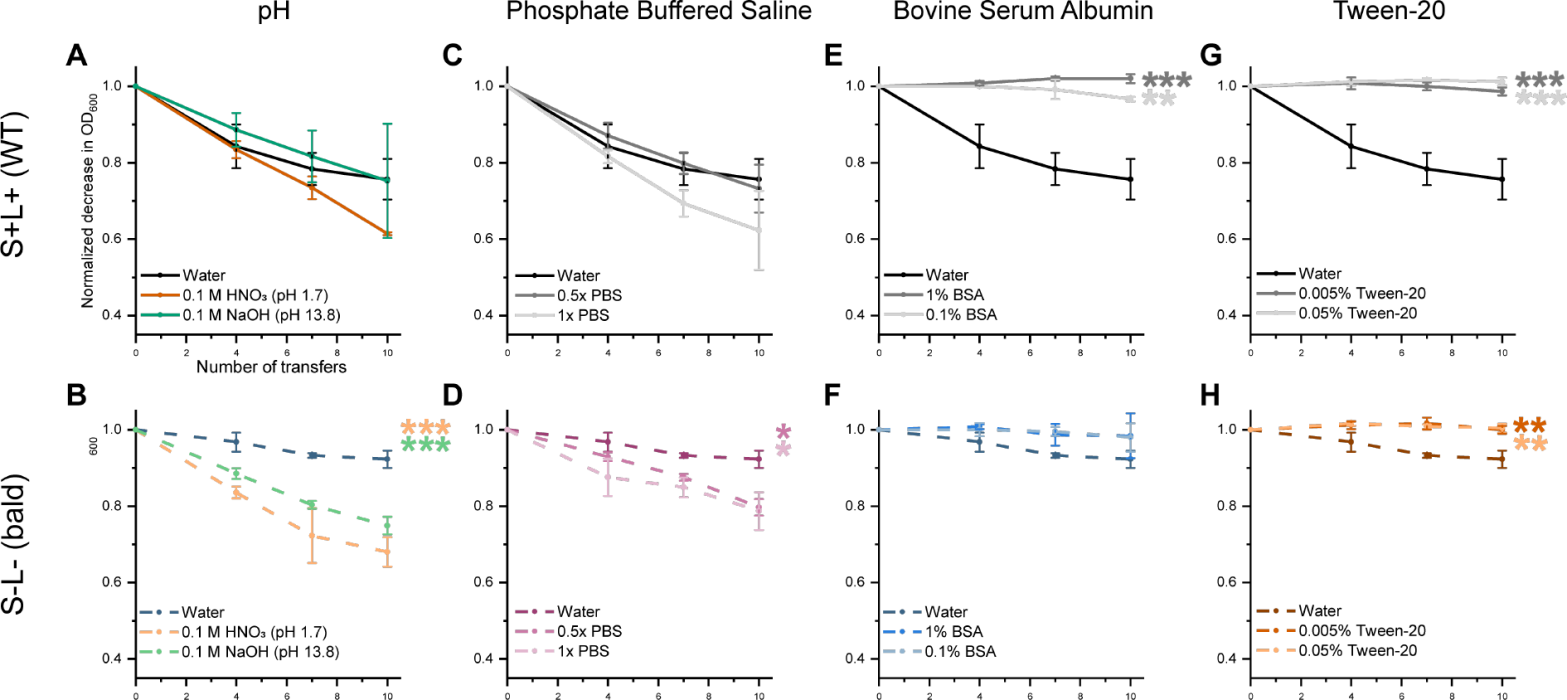
Loss of OD_600_ during sequential transfers between SS coupons is shown for WT spores (solid lines) and bald (dashed lines) spores suspended in sterile water and under different other conditions: (**A and B**) 0.1 M HNO3 (pH 1.7) and 0.1 M NaOH (pH 13.8), (**C and D**) 0.5x and 1x PBS, (**E and F**) 0.1% and 1% BSA and lastly (**G and H**) 0.005% and 0.05% Tween-20. The addition of ions, either through pH adjustment or increased salt concentration (PBS), enhanced adhesion of bald spores to the SS surface. In contrast, both BSA and Tween-20 significantly reduced adhesion for both spore types. One asterisk (*P* ≤ 0.05) indicates significant difference, while two (*P* ≤ 0.01) and three (*P* ≤ 0.001) asterisks represent increasingly stronger levels of statistical significance.

WT spores exhibited consistent adhesion to SS surfaces regardless of pH, showing no significant difference between acidic (0.1 M HNO₃, pH 1.7) and alkaline (0.1 M NaOH, pH 13.8) conditions compared to adhesion in sterile water. In contrast, bald spores displayed significantly increased adhesion under both acidic and alkaline conditions, with *P* = 0.0001 and *P* = 0.0008, respectively, after 10 transfers (Fig. 9A and B). A similar pattern was observed in phosphate-buffered saline (PBS), where bald spores adhered more effectively under elevated ionic strength (0.5× and 1× PBS) compared to sterile water, with *P* = 0.0169 and *P* = 0.0123, respectively (Fig. 9C and D). Notably, increased salt concentration did not influence adhesion of WT spores, which remained constant across all tested conditions.

In contrast to the trend observed when adding ions to the solution, the addition of BSA (Fig. 9E and F) reduced the adhesion of WT spores at both tested concentrations (*P*-values ≤ 0.001), while having no significant effect on the adhesion of bald spores. The non-ionic surfactant Tween-20 also led to a significant reduction in adhesion for both WT and bald spores at concentrations of 0.005% and 0.05% (*P* ≤ 0.005), compared to spores suspended in sterile water (Fig. 9G and H).

## DISCUSSION

In this study, we investigated the role of ENAs in spore adhesion to abiotic surfaces using a single strain of *B. paranthracis* and its isogenic mutants under various conditions. We further examined how selected environmental factors modulate spore adhesion under varying conditions. Our results showed that both WT and bald spores adhered most efficiently to PP and SS surfaces, whereas exosporium-deficient spores and vegetative cells exhibited significantly lower levels of adhesion. Moreover, spores with appendages adhered significantly more effectively to PP and SS surfaces compared to bald spores (Fig. 2B). In contrast, no statistically significant difference (*P* > 0.05) in adhesion between WT and bald spores was observed on glass and PS, indicating that ENAs did not influence attachment to these materials.

We also investigated whether the surface properties of the tested materials influenced spore adhesion by examining both the wettability of the materials as well as the roughness. PP and PS were shown to be nonpolar and hydrophobic, whereas SS and glass were identified as hydrophilic (Fig. 3). Exploring further if the observed adhesion was driven by material hydrophobicity, a spore transfer assay was conducted using dimensionally similar surfaces that differed in wettability. The results showed that neither the hydrophobic nor hydrophilic nature of the materials determined spore adhesion mediated by ENAs. The tested surfaces also differ significantly in their structure (Fig. 6A). The role of surface roughness in bacterial adhesion is complex; while some studies suggest that increased roughness facilitates adhesion by providing more contact points or protective niches, others report minimal or negligible effects (23–25). Nevertheless, there is a general agreement that rougher surfaces provide a greater surface area, offering more sites for attachment as well as larger cavities that can shield bacteria from hydrodynamic shear forces (26, 27). As shown in Fig. 6B, the SS surface is markedly rougher than the other materials tested, exhibiting surface features on a scale approximately 100-fold greater and with dominant structures significantly larger than bacterial endospores (1–2 µm). PP also had considerable surface roughness, albeit less pronounced than SS, but greater than that of glass and PS. Surface roughness measurements confirmed that both SS and PP have increased surface areas compared to PS and glass. Given that WT spores adhered more efficiently than bald spores on the two roughest materials, we hypothesize that ENAs particularly enhance spore adhesion to surfaces with rougher surface topographies.

While we can assess the role of ENAs by comparing adhesion of WT and mutant spores across different materials, we must be cautious when interpreting overall differences in spore adhesion between material types. The material properties may differ in additional ways not assessed in this study, e.g., surface charge, topological patterns, and stiffness. As our focus is specifically on the role of ENAs in adhesion, a comprehensive material characterization falls outside the scope of this work and was, therefore, not included. In addition, the substantial standard deviations observed in roughness, surface ratio, and autocorrelation measurements likely reflect inherent variability introduced during the manufacturing process of the materials and should also be considered when interpreting the results.

Consistent with previous studies, we found that spores with an exosporium adhered more efficiently to various abiotic surfaces than those with a deficient exosporium (28, 29). This enhanced adhesion is often attributed to the increased hydrophobicity of spores with an intact exosporium, which has been correlated with increased adhesion to hydrophobic surfaces (7, 13, 30, 31). However, several studies have questioned the strength of this correlation, suggesting that morphological factors, such as the presence of surface structures like ENAs, may also significantly influence spore adhesion (10, 13, 32). Our results support this view, as the hydrophobicity of the tested materials had limited impact on the adhesion of WT spores (Fig. 4).

We have previously shown that removal of S-ENAs significantly reduces the hydrodynamic diameter of the spores, thereby influencing their behavior and interactions in an aqueous environment. The presence of S-ENAs also appears to act as a steric hindrance, limiting the ability of spores to form close interactions with surfaces or other particles (17, 18). Polystyrene is an aromatic polymer composed of a linear hydrocarbon backbone with a benzene ring attached to every second carbon atom. In this study, surface-treated polystyrene was used in an attempt to isolate the effect of hydrophobicity on spore adhesion. However, the surface modification process may also affect other material properties. For instance, chemical treatments designed to enhance hydrophilicity often introduce new functional side chains, which can alter the surface’s topographical features and physicochemical characteristics (33).

Tauveron et al. (10) reported that spores with longer appendages adhere better to abiotic surfaces than those with shorter ones (10). Based on this, we hypothesized that spores expressing S-ENAs would adhere more effectively than those expressing only L-ENAs or bald spores. Consistent with this, bald spores showed significantly reduced adherence to PP and SS compared to WT spores (Fig. 7, *P* = 0.0004 and *P* = 0.0065, respectively). However, for S−L+ and S+L- spores, reduced adhesion reached statistical significance only for PP, not for SS. This suggests that the expression of either ENA type alone may be sufficient to maintain WT-like adhesion to SS, whereas the depletion of one appendage type disrupts adhesion, particularly PP. These findings underscore the complex interplay between surface material properties and bacterial surface structures and point to a complementary role of both S- and L-ENAs in facilitating efficient spore attachment to abiotic surfaces. We have previously shown that both S- and L-ENAs facilitate spore aggregation in two distinct ways: S-ENAs facilitate longer-range interaction, whereas L-ENAs mediate more intimate close-range aggregation of spores (17). In the current study, we were unable to identify any differences in adhesion behavior between the two types of appendages.

Adhesion testing on PP over different incubation times revealed the dynamic nature of spore attachment and underscored the critical role of ENAs in maintaining stable interactions with abiotic surfaces. Although no statistically significant differences were observed between the strains at individual timepoints, the overall adhesion dynamics showed that WT spores consistently exhibited the highest and most stable adhesion to PP, emphasizing the importance of ENAs in sustaining prolonged surface binding (Fig. 8). In contrast, bald spores exhibited a transient adhesion pattern, suggesting a reduced capacity to maintain long-term attachment. This likely reflects weaker initial binding forces that are insufficient to withstand extended incubation. These findings emphasize the time-dependent nature of spore adhesion and demonstrate the distinct contributions of ENAs and the exosporium to stable and sustained surface interactions. Notably, under static conditions (Fig. 7A and B), both S- and L-ENAs were seen to be required for efficient adhesion to PP, while spores lacking appendages adhered poorly. However, under dynamic conditions with extended contact time (Fig. 8), mutant spores expressing only one type of ENA adhered similar to wild type spores. This suggests that the dynamic setup, which imposes continuous mixing and prolonged interaction, may compensate for the absence of one type of fiber and promote eventual stable adhesion across all strains. The MATH assay also revealed a significantly greater batch variability in the hydrophobicity of the bald spores compared to spores of the other strains tested. This variation may stem from potential differences in the ability of bald and ENA-expressing spores to adsorb extracellular materials such as environmental DNA, polysaccharides, and proteins, likely contributing to the lack of statistically significant differences observed.

Understanding the adhesion dynamics of ENAs provides crucial insights into how spores interact with surfaces, but adhesion is also influenced by various external factors in the surrounding solution. For instance, WT spores suspended in surfactants (e.g., Tween-20), or in solubilized protein (e.g., BSA) showed reduced adhesion compared to spores suspended in water (Fig. 9E through H). This could be attributed to the blocking of binding sites on both the spores and the substrate surfaces, which limits interactions and thereby lowers adhesion as demonstrated by our observations. We also showed that the presence of PBS in the spore suspension resulted in a concentration-dependent reduction in both ENA- and exosporium-mediated spore-aggregation (17). The reduction was more pronounced for WT spores than for bald spores, indicating that appendage-mediated spore aggregation is more sensitive to increased ionic strength than aggregation primarily mediated by the exosporium (17). In the present study, we observed that the adhesion efficiency of bald spores to SS increased under conditions of elevated ionic strength (PBS, 0.1 M HNO₃, and 0.1 M NaOH) (Fig. 9A through D), whereas the adhesion of WT spores remained largely unaffected. These results are consistent with previous studies reporting enhanced spore adhesion to SS in high ionic strength conditions. This effect is thought to occur because, under high salt or acidic conditions, water is drawn away from the spore surface. This “dehydration” of the spore’s outer layer reduces the natural repelling forces that usually keep spores and surfaces apart in liquid. With these barriers weakened, spores can get closer to the surface, leading to stronger adhesion (34). In addition, bacterial appendages, like ENAs, can facilitate bacterial adhesion, as they are able to reach through the repulsive forces of the electrostatic double layer and make direct contact with the surface (35). This effect might explain why WT spores showed stronger and more stable adhesion in our experiments. In a previous study, we used an optical tweezer system and found that WT spores could be moved along a surface, but not lifted off, suggesting that they were tethered by their appendages (18). The same study also showed that L-ENAs are especially important for anchoring spores to surfaces. In contrast, bald spores, which lack these appendages, do not have the same ability to attach directly to surfaces. This likely explains their weaker initial adhesion, especially to stainless steel (SS). However, when the ionic levels increase and the electrical double layer is compressed, the spore-surface might come into closer proximity to the material surface, promoting adhesion through other mechanisms than surface appendages (35).

Acidic and alkaline washes are commonly used in cleaning-in-place procedures to remove biological material residues from pipes and processing equipment. However, our findings suggests that adhesion of bald spores increases under both acidic and alkaline conditions compared to pure water, while WT spores consistently maintain strong adhesion across all conditions. This sustained surface adhesion may reduce the effectiveness of industrial cleaning processes, as surface-adhered spores tend to have an enhanced tolerance to alkali compared to spores remaining in suspension (36). In real-world processing environments, spores are often introduced through complex media, like milk, which contains a variety of components such as salts, lipids, and proteins. Our results show that spore adhesion is strongly influenced by the surrounding liquid environment. Specifically, spores suspended in surfactants (Tween-20) or in solubilized protein (BSA) showed reduced adhesion, whereas higher ionic strength promoted stronger attachment. To effectively reduce spore adhesion during processing, more knowledge is needed about how spores behave in complex food matrices like milk. Understanding which factors limit or promote initial adhesion will be crucial for developing improved cleaning strategies and preventing contamination.

In conclusion, this study has specifically investigated the role of ENAs in spore adhesion to abiotic surfaces by comparing WT spores, a panel of isogenic mutant spores, and vegetative cells. Our findings demonstrate that ENAs play a significant role in enhancing spore adhesion to materials such as PP and SS. While surface hydrophobicity did not appear to be a major factor influencing ENA-mediated spore adhesion, surface properties such as roughness emerged as more influential. The importance of understanding spore adhesion is underscored by the fact that attached spores exhibit markedly increased resistance to various stressors. For instance, adhered spores can display increased alkali tolerance and up to a 400% increase in heat tolerance compared to non-adhered spores (31, 36). Such resilience poses challenges in both food processing environments and clinical settings, where spore contamination can lead to significant safety and hygiene concerns. Gaining a deeper understanding of the factors influencing spore adherence is critical for developing targeted strategies to mitigate spore contamination. This knowledge is essential for designing effective cleaning protocols, improving surface materials to reduce spore adhesion, and ultimately minimizing the risks associated with spore persistence in sensitive environments.

## MATERIALS AND METHODS

### Spore preparations

Spores of the *B. paranthracis* strain NVH 0075/95 (20) and its isogenic mutants with different appendage compositions were used in this study in addition to an NVH 0075/95 Δ*exsY* mutant with a deficient exosporium. Comparing multiple strains enabled us to isolate the specific effects of the appendages, offering deeper insight into their role in adhesion to abiotic surfaces. The genotypes and corresponding phenotypes of all mutant strains are presented in Table 1.

The spores were prepared in a sporulation media containing 8 g/L Nutrient Broth (Oxoid, Cat. No: CM0001, Thermo Scientific), 5 mM (NH_4_)_2_SO_4_, 1 mM MgCl_2_, 1 mM Ca(NO_3_)_2_, 1 µM FeSO_4_, 66 µM MnSO_4_, 12.5 µM ZnCl_2_, 2.5 µM CuCl_2_, 2.5 µM Na_2_MoO_4_, and 2.5 µM CoCl_2_, as adapted from reference 37. The cultures were incubated at 37°C in a conical flask shaking at 200 rpm. Spores were harvested when ≥ 90% of the vegetative cells had sporulated, as determined by phase-contrast microscopy. This was followed by an initial 10 min centrifugation at 3,700 × *g* (Heraeus Megafuge 16R Centrifuge equipped with a TX-400 rotor, Thermo Scientific), followed by three washes with sterile water (4°C). The spores were stored at 4°C in sterile water until use.

Fresh cultures of *B. paranthracis* were prepared by adding 1 mL of an LB overnight culture to 9 mL fresh LB media and incubated for 3 h at 37°C. The vegetative cells were harvested using the same procedure as for the spores, albeit with centrifugation at 2,500 × *g* for 10 min.

### Adhesion experiments

Spore adhesion testing was performed using a modified version of the method described by (38). Initially, the number of transfers was determined to be 10 based on the measurement of how many transfers were required to reach an OD of 0.5 for WT in a polystyrene cuvette. The spore concentration in a sterile water suspension was adjusted to 0.8 by measuring the optical density at 600 nm (OD_600_). A volume of 1 mL of the OD adjusted spore suspension was transferred to a PS cuvette (BR75901 5, Brand) using a Pasteur pipette (No. 612-1702, VWR), and the OD_600_ was measured again. Pasteur pipettes were used as the spores exhibited lower adherence to the glass material of these pipettes, compared to plastic pipette tips, as determined by visual inspection (data not shown). This procedure was repeated nine additional times, with the spore suspension sequentially transferred to new cuvettes and OD_600_ recorded after each transfer.

The decrease in OD_600_ was used as an indirect measure of the number of spores that adhered to the vial surfaces. The following formula was used to calculate the proportion of non-adherent spores remaining in the suspension: OD_*n*_/OD_*i*_, where OD_*i*_ is the initial OD_600_ and OD_*n*_ is the OD_600_ after *n* transfers. A similar procedure was also used to analyze spore adhesion to glass, SS, and PP tubes. Here, we used glass test tubes (14 mm × 130 mm, DWK Life Sciences), 316 stainless steel pipe fittings (No. 499-3467, RS PRO), and 1.5 mL microcentrifuge tubes of PP (REF 72.690.001, Sarstedt). Adhesion by vegetative cells was assessed using the same procedure as for spores for all materials tested. Lastly, the same setup was used to investigate how phosphate-buffered saline (PBS), BSA (1%, A6003, Sigma-Aldrich), and Tween-20 (0.05%, BP337-100, Fisher BioReagents) affected adhesion to SS by suspending the spores in the respective solutions instead of sterile water.

### Assessing adhesion over time

The WT and isogenic mutant strains were prepared as described in the adhesion experiment section, with the initial OD_600_ adjusted to 0.8. To observe the effect of adhesion over time, the tubes were placed in a HulaMixer (15920D, Thermo Scientific) set to rotate at 40 rpm. They were allowed to rotate for either 1 min, 10 min, 30 min, or 1 h. After each time point, the tubes were removed from the mixer, and OD_600_ was measured. Calculations were performed as described in the previous section.

### Microtiter experiments

To compare the role of material wettability on spore adhesion, we used microtiter plates to ensure uniform measurements. The plates used for this analysis included a PS Nunclon Delta-treated plate (168055, Thermo Scientific) representative of hydrophilic surface-treated polystyrene, and a hydrophobic COSTAR PS-untreated plate (Corning Polystyrene Not Treated, Cat. no. CLS3370, Corning, Inc.). The OD_600_ of the spore suspension was adjusted to 0.8, and 200 µL was transferred to the first well. After pipetting the spore suspension into the first well, the plate was loaded into the Infinite M200 microplate reader (Tecan Trading AG). The OD_600_ of the sample was recorded, after which the spore suspension was transferred 3, 6, and 9 times using Pasteur pipettes. Calculations were performed as described in the “dhesion experiments” section.

### Microbial adhesion to hydrocarbon

The relative hydrophobicity of spores was estimated using the microbial adhesion to hydrocarbon (MATH) assay, as described previously 28, 39. The spore suspension was diluted to an OD_600_ of approximately 1.6 in glass test tubes (14 mm × 130 mm, DWK Life Sciences), and a volume of 1.5 mL of hexadecane (99% purity, H6703, Sigma-Aldrich) was added to 2 mL of the spore suspension. The tubes were vigorously vortexed for 1 min and left to settle at 30°C before being vortexed for another 2 min. The phases were allowed to separate at room temperature for 20 min before the OD_600_ of the bottom aqueous phase was measured.

The percentage of relative hydrophobicity was calculated using the following formula:


$$Relative\;hydrophobicity=\;\frac{{OD}_{600}\;Start-{OD}_{600}\;End}{{OD}_{600}\;Start}\times 100$$

### Contact angle measurements

Contact angle measurements were performed using a Theta One tensiometer (Attension, Biolin Scientific). A 4 µL drop of deionized water was generated above the material surface using a gauge 22 needle (C209-22) at a rate of 1 µL/s. A total of 150 image frames were acquired over 10 s immediately after the drop was deposited and the contact angle was determined.

### Surface roughness analyses

Surface topography was characterized using AFM or stylus profilometry, depending on sample type. AFM scans (100 µm²) were acquired in tapping mode using a Park NX-Hivac (Park Systems Corp.) system with a PPP-NCHR (Park Systems Corp.) probe (tip radius < 10 nm). SS surfaces were instead scanned using a Dektak XT stylus profilometer (Bruker) over 1 mm² areas with 10 µm lateral resolution, to better capture larger-scale surface features. Surface maps were processed in Gwyddion (v. 2.67) (40) in order to minimize imaging artifacts, using sample-specific background correction procedures.

We quantified three key surface parameters, as an average of three measurements from different samples (two for glass): Root mean square (RMS) roughness, reflecting vertical height variation, surface ratio (SR = surface area/projected area), indicating topographical expansion, and autocorrelation length (ACL), describing the lateral scale over which surface features remain correlated. ACL was estimated using two complementary approaches. First, an orientation-independent ACL was derived from the 2D power spectral density function (PSDF). Second, directional ACLs were evaluated along horizontal and vertical axes using Gwyddion’s correlation length tool. In both cases, the ACL was estimated based on the distance at which the autocorrelation function decays to 1/e of its maximum.

### Electron microscopy

Scanning electron microscopy was used to observe spore adhesion on SS, PP, PS, and glass surfaces. The materials were cut into smaller pieces suitable for SEM analysis and then immersed in spore suspension (OD_600_ = 0.8) prepared in sterile water. After 1 h of incubation, the spore suspension was fixed with a 4% formaldehyde solution, which was applied for 15 min. The spore sample was then rinsed three times with sterile water and left to dry overnight. Subsequently, the samples were coated with an approximately ~30 nm layer of platinum using a sputter coater (Leica EM ACE200). Images were captured using a Zeiss EVO 50 scanning electron microscope operating in high vacuum mode with an accelerating voltage of 10 kV and a current of 20 pA and at a magnification of 3,000×.

### Data analysis and statistics

Statistical analysis was performed using Prism (Prism 10.3, GraphPad). One-way ANOVA was applied to the data from the 10th transfer in each experiment series, followed by Dunnett’s (Fig. 2, 5 and 7 to 9) multiple comparisons test to check for differences relative to the WT strain, or Tukey’s (Fig. 4) multiple comparisons test to assess differences between all strains. Statistical significance is indicated as follows: *P* > 0.05 (ns), *P* ≤ 0.05 (*), *P* ≤ 0.01 (**), *P* ≤ 0.001 (***), and *P* ≤ 0.0001 (****). All *P*-values are summarized and available in the supplemental material under Statistical analysis results, Table S6 to S11. Graphing and data visualization were performed using Origin 2024 (OriginLab).

## SUPPLEMENTAL MATERIAL

Supplemental material aem.00944-25-s0001.pdf Data from contact angle and surface roughness measurements and statistical analyses.

ASM does not own the copyrights to Supplemental Material that may be linked to, or accessed through, an article. The authors have granted ASM a non-exclusive, world-wide license to publish the Supplemental Material files. Please contact the corresponding author directly for reuse.
