# Supplementary material for: Endospore appendages enhance adhesion of Bacillus cereus sensu lato spores to industrial surfaces, modulated by physicochemical factors
Source: Appl Environ Microbiol. 2025 Oct 7;91(11):e00944-25. doi: 10.1128/aem.00944-25 (PMC12628813; doi:10.1128/aem.00944-25)
Supplement: Supplemental material — Data from contact angle and surface roughness measurements and statistical analyses. [file aem.00944-25-s0001.pdf]

Supplementary Information

Endospore appendages enhance adhesion of *Bacillus cereus sensu lato* spores to industrial surfaces, modulated by physicochemical factors

Unni Lise Albertsdottir Jonsmoen[1*], Jennie Ann Allred[1*], Dmitry Malyshev[2], Jonas Segervald[2], Magnus Andersson[2,3*] and Marina Elisabeth Aspholm[1*].

[1] Department of Paraclinical Sciences, Faculty of Veterinary Medicine, Norwegian University of Life Sciences (NMBU), Ås, Norway

[2] Department of Physics, Umeå University, Umeå, Sweden

[3] Umeå Centre for Microbial Research (UCMR)

* Co-first authors contributed equally to this work. The order of authorship was determined based on Unni Lise Jonsmoen's initiation of the study

* Corresponding authors

E-mail: marina.aspholm@nmbu.no (MEA) and magnus.andersson@umu.se

Contents

Data for contact angle and surface roughness measurements *2*

Limited role of surface and spore hydrophobicity in spore adhesion (Figure 3) 2

Figure S1: Image frames as captured from the Theta One tensiometer 2

Table S1: Measurements acquired using the Theta One tensiometer 2

Stainless steel features large surface structures, while glass exhibited overall lowest roughness (Figure 6) 3

Table S2: Summarised output from profilometer for stainless steel 3

Table S3: Summarised output from atomic force microscopy for polypropylene 3

Table S4: Summarised output from atomic force microscopy for polystyrene 3

Table S5: Summarised output from atomic force microscopy for glass 3

Statistical analysis results *4*

Table S6: Summary of *P*-values from data presented in Figure 2 4

Table S7: Summary of *P*-values from data presented in Figure 4 5

Table S8: Summary of *P*-values from data presented in Figure 5 5

Table S9: Summary of *P*-values from data presented in Figure 7 5

Table S10: Summary of *P*-values from data presented in Figure 8 6

Table S11: Summary of *P*-values from data presented in Figure 9 6

Data for contact angle and surface roughness measurements

The wettability of stainless steel (SS), polypropylene (PP), polystyrene (PS) and glass was determined using a tensiometer, which measured the angle formed between a water droplet and the surface material. The material could therefore be classified as either hydrophobic (> 90°) or hydrophillic (< 90°). An in-depth material characterisation was also undertaken, using a profilometer and atomic force microscope. This allowed us to quantify the material's roughness, surface ratio, as well as Fourier, vertical, and horizontal autocorrelation parameters.

Limited role of surface and spore hydrophobicity in spore adhesion (Figure 3)

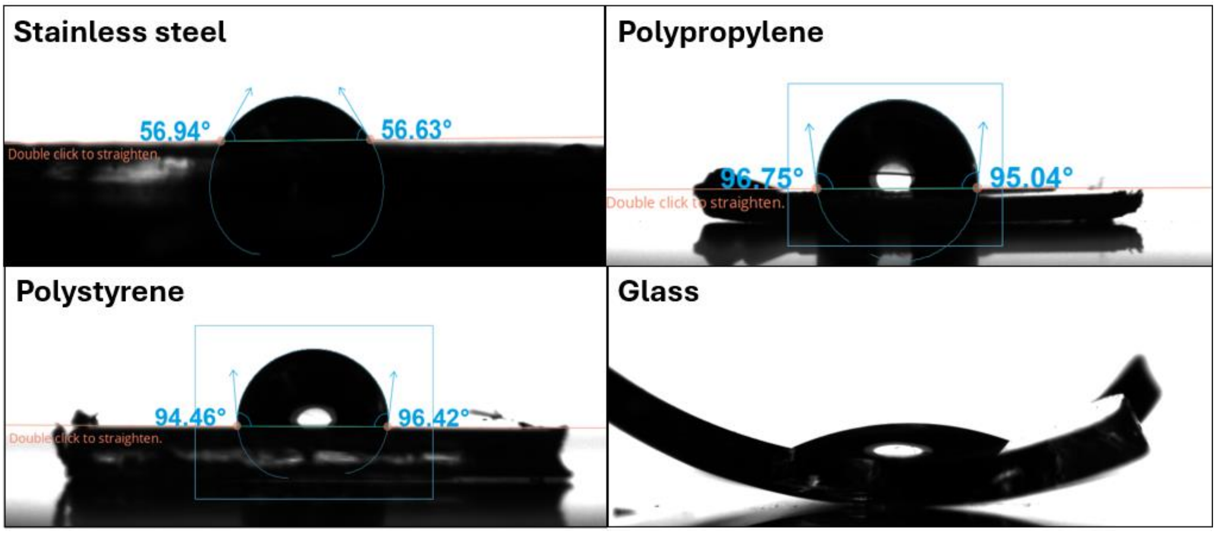

Figure S1: Examples of image frames, captured from the Theta One tensiometer (Attension®, Biolin Scientific), for stainless steel, polypropylene, polystyrene and glass. A total of 150 image frames over 10 seconds were captured for each material.

Table S1: Measurements acquired using the Theta One tensiometer, with mean values and standard deviations displayed for each tested material

**Material**	**Average (°)**	**Standard deviation (°)**
SS	64.8	10.4
PP	97.9	4.2
PS	92.3	3.9
Glass	-	-

Stainless steel features large surface structures, while glass exhibited overall lowest roughness (Figure 6)

Table S2: Summarised output from profilometer for stainless steel

	Stainless steel				
			Autocorrelation		
	RMS roughness (μm)	*Surface ratio*	*Fourier transform (μm)*	*Vertical (μm)*	*Horizontal (μm)*
Average	7.02	1.02	60.29	101.20	113.97
Standard deviation	0.38	0.00	8.45	4.42	17.39

Table S3: Summarised output from atomic force microscopy for polypropylene

	Polypropylene				
			Autocorrelation		
	RMS roughness (nm)	*Surface ratio*	*Fourier transform (μm)*	*Vertical (μm)*	*Horizontal (μm)*
Average	91.85	1.02	5.92	8.51	1.25
Standard deviation	19.79	0.00	3.39	2.62	0.36

Table S4: Summarised output from atomic force microscopy for polystyrene

	Polystyrene				
			Autocorrelation		
	RMS roughness (nm)	*Surface ratio*	*Fourier transform (μm)*	*Vertical (μm)*	*Horizontal (μm)*
Average	29.18	1.00	7.48	7.05	5.99
Standard deviation	15.71	0.00	2.63	4.30	3.86

Table S5: Summarised output from atomic force microscopy for glass

	Glass				
			Autocorrelation		
	RMS roughness (nm)	*Surface ratio*	*Fourier transform (µm)*	*Vertical (µm)*	*Horizontal (µm)*
Average	5.15	1.00	2.24	0.42	0.59
Standard deviation	1.73	0.00	2.74	0.22	0.30

Statistical analysis results

One-way ANOVA was applied to the data from the 10th transfer in each experiment series, followed by Dunnett's (Figure **2, 5, 7, 8** and **9**) multiple comparisons test to check for differences relative to the WT strain, or Tukey's (Figure **4**) multiple comparisons test to assess differences between all strains.

Table S6: Summary of *P*-values from data presented in Figure 2*,* with the mean of WT pairwise compared to the mean of each other strain.

Strains		Material	***P*-values**
S+L+ (WT)	S-L-(bald)		0.0014
	ΔexsY		0.0003
	Vegetative cells	*SS*	0.0003
	S-L-(bald)		0.0004
	ΔexsY		0.0002
	Vegetative cells	*PP*	0.0002
	S-L-(bald)		0.799
	ΔexsY		0.0002
	Vegetative cells	*PS*	0.0005
	S-L-(Bald)		0.6808
	ΔexsY		0.0005
	Vegetative cells	*Glass*	0.0002

Table S7: Summary of *P*-values from data presented in Figure 4, with the mean of each condition compared to the mean of every other condition

**Pairwise comparison**	***P*-values**
WT PS-untreated vs. bald PS-untreated	> 0.9999
WT PS-untreated vs. WT PS-treated	0.1891
WT PS-untreated vs. bald PS-treated	0.0482
bald PS-untreated vs. WT PS-treated	0.1847
bald PS-untreated vs. bald PS-treated	0.047
WT PS-treated vs. bald PS-treated	0.7663

Table S8: Summary of *P*-values from data presented in Figure 5, with the mean of WT pairwise compared to the mean of each other strain.

**Strains**		***P*-values**
S+L+ (WT)	S-L-(bald)	0.4247
	S+L-	0.9999
	S-L+	0.9826
	ΔexsY	< 0.0001

Table S9: Summary of *P*-values from data presented in Figure 7, with the mean of WT pairwise compared to the mean of each other strain.

**Strains**		**Material**	***P*-values**
S+L+ (WT)	S-L- (bald)	*SS*	0.0065
	ΔexsY		0.0009
	S-L+		0.4313
	S+L-		0.1685
	Vegetative cells		0.0013
	S-L- (bald)	*PP*	0.0004
	ΔexsY		0.0001
	S-L+		0.0031
	S+L-		0.0114
	Vegetative cells		0.0001

Table S10: Summary of *P*-values from data presented in Figure 8, with the mean of WT pairwise compared to the mean of each other strain.

Strains		Material	*P*-values
S+L+ (WT)	S-L- (bald)	*PP*	0.0939
	ΔexsY		0.0803
	S-L+		0.4115
	S+L-		0.8127

Table S11: Summary of *P*-values from data presented in Figure 9, with the mean of WT and bald in water pairwise compared to the mean of each of the conditions (pH, PBS, BSA, Tween-20)

Strains		Material	*P*-values
Water			
S+L+ (WT)	0.1 M HNO_3	*pH*	0.1891
	0.1 M NaOH		0.9980
S-L- (bald)	0.1 M HNO_3		0.0001
	0.1 M NaOH		0.0008
S+L+ (WT)	0.5x	*PBS*	0.9282
	1x		0.2148
S-L- (bald)	0.5x		0.0169
	1x		0.0123
S+L+ (WT	0.1%	*BSA*	0.0010
	1%		0.0003
S-L- (bald)	0.1%		0.3612
	1%		0.3186
S+L+ (WT	0.05%	*Tween-20*	0.0004
	0.005%		0.0006
S-L- (bald)	0.05%		0.0038
	0.005%		0.0050
